# Alterations of Voltage-Dependent Calcium Channel Currents in Basilar Artery Smooth Muscle Cells at Early Stage of Subarachnoid Hemorrhage in a Rabbit Model

**DOI:** 10.1371/journal.pone.0084129

**Published:** 2014-01-02

**Authors:** Xianqing Shi, Yongjian Fu, Daqing Liao, Yanfang Chen, Jin Liu

**Affiliations:** 1 Intensive Care Unit, Guizhou Province People's Hospital, Guiyang, China; 2 Laboratory of Anesthesia and Critical Medicine, West China hospital, Sichuan University, Chengdou, China; National Institutes of Health, United States of America

## Abstract

**Objective:**

To investigate the changes in the currents of voltage-dependent calcium channels (VDCCs) in smooth muscle cells of basilar artery in a rabbit model of subarachnoid hemorrhage (SAH).

**Methods:**

New Zealand white rabbits were randomly divided into five groups: sham (C), normal (N), 24 hours (S1), 48 hours (S2) and 72 hours (S3) after SAH. Non-heparinized autologous arterial blood (1ml/kg) was injected into the cisterna magna to create SAH after intravenous anesthesia, and 1 ml/kg of saline was injected into cisterna magna in the sham group. Rabbits in group N received no injections. Basilar artery in S1, S2, S3 group were isolated at 24, 48, 72 hours after SAH. Basilar artery in group C was isolated at 72 hours after physiological saline injection. Basilar artery smooth muscle cells were isolated for all groups. Whole-cell patch-clamp technique was utilized to record cell membrane capacitance and VDCCs currents. The VDCCs antagonist nifedipine was added to the bath solution to block the Ca^++^ channels currents.

**Results:**

There were no significant differences in the number of cells isolated, the cell size and membrane capacitance among all the five groups. VDCC currents in the S1–S3 groups had higher amplitudes than those in control and sham groups. The significant change of current amplitude was observed at 72 hours after SAH, which was higher than those of 24 and 48 hours. The VDCCs were shown to expression in human artery smooth muscle cells.

**Conclusions:**

The changes of activation characteristics and voltage-current relationship at 72 hours after SAH might be an important event which leads to a series of molecular events in the microenvironment of the basilar artery smooth muscle cells. This may be the key time point for potential therapeutic intervention against subarachnoid hemorrhage.

## Introduction

When a cerebral aneurysm ruptures, bleeding and clot formation occur around the surface of the brain, including several major blood vessels. The resulting condition, known as subarachnoid hemorrhage (SAH) often results in death or severe disability and is a significant cause of stroke. Cerebral vasospasm (CVS) and the resulting cerebral ischemia occurring after subarachnoid hemorrhage (SAH) are responsible for the considerable morbidity and mortality [1]. The overall mortality rates range from 32% to 67% with 10–20% of patients with long-term dependence due to brain damage [2]. Mechanisms contributing to the development of vasospasm have been investigated in recent years and the studies demonstrated that CVS have relationship with the disorder of ion channels in cerebral vessels after SAH [3],[4],[5]. The voltage-dependent calcium channels (VDCCs) might have important role in CVS because they could regulate the intracellular calcium concentration and contraction of cerebral vessels [6], [7], [8], [9]. VDCCs serve as a main potential-dependent Ca^2+^ entry pathway in a wide range of tissues/cell types and have been implicated in a variety of cellular processes including oxidative stress, mitochondrial dysfunction and cell death in neuronal dysfunction [10], [11], [12].

Five types of voltage-dependent calcium channels are known (namely L-,T-, N-, P/Q- and R-types) and have different voltage ranges of activation, single channel conductance and sensitivities to pharmacological agents [13]. L-, N-, P/Q- and R-type voltage-dependent calcium channels tend to be activated at less negative voltages and thus have been referred to as high voltage activated (HVA) calcium channels whereas T-type channels activate at lower potentials and have been called low voltage-activated (LVA) channels. Expression of different types of voltage-dependent calcium channels in vascular smooth muscle appears to vary with species, physiological and pathological conditions. Basilar artery smooth muscle cells are shown to be heterogeneous in terms of types of voltage-dependent calcium channel currents, which are not well characterized [14], [15]. Here, we described voltage-dependent calcium channel currents in rabbit basilar artery smooth muscle cells following SAH. We showed that these cells, in addition to HVA channels, possess LVA calcium channels of the T-type and that they have LVA calcium channel currents. Previous studies have demonstrated that the protein of voltage-dependent calcium channels had alteration in basilar artery smooth muscle cells after SAH [16], [17]. The role of the voltage-dependent calcium channels has been studied at later stages in a dog model after SAH [18]. Here we demonstrated that the change of VDCC currents occurred at an early period up to 72 hours in rabbit SAH model.

## Materials and Methods

### Ethics statement

The animal experimental protocols were approved by the Animal Care and Research Committee of Sichuan University (Approval No. SCXK092012). All procedures were conducted in accordance with the Guidance Suggestions for the Care and Use of Laboratory Animals, formulated by the Ministry of Science and Technology of the People's Republic of China [19]. All surgery was performed under anesthesia, and all efforts were made to minimize sufferings.

### Reagents

Papain, dithioerythritol, XI type collagenase, Na2-ATP, TEA-Cl and CsCl were purchased from Sigma-Aldrich (USA).

### Design of subarachnoid hemorrhage model in rabbit

It was randomized, double-blinded and controlled study. New Zealand white rabbits weighing 2.5–3.0 Kg were randomly assigned to normal group (N), sham group (C), 24 hours (S1), 48 hours (S2) and 72 hours (S3) after SAH (n = 8).The animals were supplied by the Animal center of China West Hospital of Sichuan University.

The study adopted a previously described rabbit SAH model [20]. Briefly, under pentobarbital (1 ml/kg) and midazolam (1 ml/kg) intravenous anesthesia, a midline incision was made in a prone position to expose the at lanto-occipital membrane. 1 ml/kg non-heparinized autologous arterial blood was obtained from the central ear artery and injected into the cisterna magna in S1∼S3 group. Saline (at 37°C) was injected into cisterna magna in C group rabbits, whereas nothing was done in N group. Subsequently, the rabbits in S1–S3 group were positioned in ventral recumbence for 30 minutes to allow for ventral blood clot formation. Heart rate, respiration rate, pulse oxygen saturation, and rectal temperature were monitored during surgery, and rectal temperature was maintained at 37–38°C. Normal saline (0.9% Sodium chloride) were administered to the animals with a peristaltic pump at a rate of 20 ml/min. Penicillin (200 000 U/kg) was intravenously injected following surgery. The rabbits were housed in cages following suturing and sterilizing of the incision. The animals were observed for 72 hours in standard animal facilities. They had free access to water and food.

### Isolation of basilar artery smooth muscle cells

Rabbits were anesthetized with an intravenous injection of sodium pentobarbital (50 mg/kg) and sacrificed at 24, 48 and 72 hours respectively following SAH in S1, S2 and S3 group. Rabbits in N and C group were anesthetized and sacrificed at 72 hours following SAH.

The basilar arteries were harvested, the endothelia were removed, the arachnoid and perivascular connective tissue were trimmed off, and the tissues were cut into 1mm square pieces. The smooth muscle cells of artery were isolated by enzymatic digestion according to previously described methods [18], [21]. Briefly, the small pieces of basilar artery was put into a glass dish containing the enzyme I solution [0.5 mg/ml papain and 1 mg/ml dithioerythritol in PPS solution (140 mM NaCL, 5.0 K mM CL, 1.3 mM MgCL2, 10 mM HEPES, 10 mM glucose, Osmolarity 300 mOsm/L and pH 7.4)] in a 37°C water bath for thirty-five minutes. Subsequently, the enzyme I solution was removed and the enzyme II solution (1 mg/ml XI type collagenase in PPS solution) was added and continued digestion in water bath at 37°C for 25 minutes. Then, the cell suspension was gently mixed by using a Pasteur pipette for 10 minutes in order to release the smooth muscle cells. The cell suspensions were persevered at 4°C for 2 hours prior to record the currents.

### Whole-cell Patch clamp recordings

Smooth muscle cells of basilar artery were visualized in a chamber with an inverted microscope (Olypmus, IX70).The pipettes was pulled from borosilicate glass tubing with a micropipette puller (Sutter Instrument Corp, P-97) and was fire polished (World Precision Instruments).They had resistances of 4–5 MΩ when filled with a pipette solution (135 mM CsCl, 3 mM MgCl2, 10 mM EGTA, 10 mM HEPES, 3 mM Na2-ATP,Osmolarity 290–300mOsm/L and pH 7.3). Whole-cell voltage-clamp was performed using a current and voltage clamp system (Multiclamp 700A,16-bit data acquisition system Digidata 1332A, Axon Instruments) driven by Multiclamp Commander (Sutter Instrument Co,MP-285). The VDCC currents were recorded using Ba2+ as charge carrier (IBa) in the bath solution (125 mM NaCL, 10 mM BaCL2, 10 mM HEPES, 10 mM glucose,125 mM TEA-CL,1 mM MgCL2. Osmolarity 290–300mOsm/L and PH 7.3). Currents through HVA and LVA VDCCs were evaluated by several voltage protocols. The smooth muscle cells were held at holding potentials (Vh) of −50 or −90mV and subjected to step depolarization of 200 msec to +80mV in 10-mV increments every 5 seconds. Data were collected after the whole-cell configuration was obtained and current amplitude had stabilized, usually 5 minutes after rupture of the cell membrane. Only cells with an input resistance greater than 1 GΩ and no run-down were analyzed. Current traces were filtered at 1 KHz with a low-pass 4-pole Bessel filter in clamp amplifier, subjected to P/4 leak subtraction and digitized at 5KHz.The currents were total voltage-dependent calcium channel's current (T-VDC) at Vh of −90mV, and were L type voltage-dependent calcium channel's current(L-VDC) at Vh of −50mv [18], [22], [23]. Voltage dependency of I_Ba_ inactivation was investigated using a double-pulse protocol with a 250 msec conditioning voltage step to potentials between −100 and +30mV in 10mV increments every 10 msec. Inactivation protocols were run without leak subtraction. The effect of Ca^++^channel antagonists were tested by using one 250-msec voltage step −20mv from Vh of −90mv for LVA and from −50mv to +30mv for HVA current. Nifedipine (1 umol/L) was applied to the bath solution to confirm the ordered currents were calcium channels currents in this study.

The currents data were recorded and collected in Clampex10.0 software and analyzed in Clampfit10.0, origin8.6 and SPSS18.0 software. The curves of current-voltage (I-V) at Vh of −50mv and −90mv, steady-state activation and inactivation were generated using the software. The voltage and slope factor under half activation and half inactivation state were obtained using the Boltzman equation nonlinear matching [22], [24], [25].

### Culture of Human Brain Vascular Smooth Muscle Cells (HBVSMC)

Primary culture of Human Brain Vascular Smooth Muscle Cells (HBVSMC) were purchased from Sciencell Research Lab (Carlsbad, CA) and cultured in recommended media at 37°C in an atmosphere of 95% air and 5% CO2.

### Real-time PCR of calcium channel genes

Total cellular RNA was extracted from HBVSMC according to the manufacturer's protocol (Qiagen RNeasy Mini Kit, Qiagen, Hilden, Germany). Total RNA was subsequently converted into cDNA by Qiagen Reverse Transcription Kit (Qiagen, Hilden, Germany). Real-time PCR was carried out using the 7500 Real-Time PCR system (Applied Biosystems) under standard conditions with 200 pM PCR primers. Each sample was analyzed in triplets using SYBR green (Applied Biosystems) as fluorescent detector and GAPDH as endogenous control. All primers were obtained from RT qPCR Primer assay kits (SAB Bioscience).

### Cell migration assay

Cell migration was determined using a ‘scratch’ wound healing assay. HBVSMCs were grown to confluence on gelatin coated 35 mm dish; the cell monolayer was scraped with a pipit tip to create a cell-free zone. The dish was washed once with the culture medium. The monolayer was then stimulated using VEGF 20 ng/ml in 0.5 FBS medium for 24 h. HBVSMC migration was quantified by measuring (1) the number of cells which migrated into the wounded area, and (2) the distance traveled by the cell front into the wounded area. Measurements were taken from three fields randomly along the culture wound. Experiments were performed in duplicate and then repeated three times. For inhibition study, cells were pretreated with or without nifedipine.

### Statistical analysis

All results were shown as mean ± SD. Intragroup differences were compared utilizing paired t-test, and intergroup differences were compared by analysis of variance. P value of 0.05 was considered statistically significant.

## Results

### Absence of physiological variables of experimental animals in each group

There were lots of blood clots around basilar artery in S1–S3 group animals after SAH. There was no loss of animals and fifty six rabbits were included in the final analyses. The weight of rabbits were no significant differences among different groups before the experiment (P>0.05). Compared with pre-experiment, the weight of rabbits decreased after SAH in group S1-S3 group (P<0.01). There were no significant differences in heart rate (HR) and Mean arterial pressure (MAP) in all groups prior to and at 30 minutes after SAH (P>0.05) (Table 1).

**Table 1:** The comparison of weight (kg), heart rate (bpm) and mean arterial pressure (mmHg).

Group	n	Pre-experiment			Post-experiment		
		Pre-Weight (Kg)	Pre-HR (bpm)	Pre-MAP (mmHg)	Post-Weight (Kg)	Post-HR (bpm)	Post-MAP (mmHg)
C	8	2.68±0.17	247±10	94±4	2.68±0.21	253±14	93±7
N	8	2.66±0.21	246±11	94±5	2.67±0.19	247±13	94±10
S1	8	2.69±0.16	249±24	95±2	2.52±0.22^d^	244±26	94±2
S2	8	2.63±0.21	249±15	95±6	2.42±0.16^abd^	243±14	93±4
S3	8	2.67±0.22	249±27	95±5	2.49±0.24^abd^	244±25	94±7

^a^ *P*<0.05 *vs.* C group, ^b^ *P*<0.05 *vs*.N group,^ d^ *P*<0.05 vs Pre-experiment, MAP: mean arterial pressure; HR: heart rate, 1 mm Hg  = 0.133 kPa.

### Consistency in Cell isolation and Cell membrane capacitance among procedures

**Figure 1:**
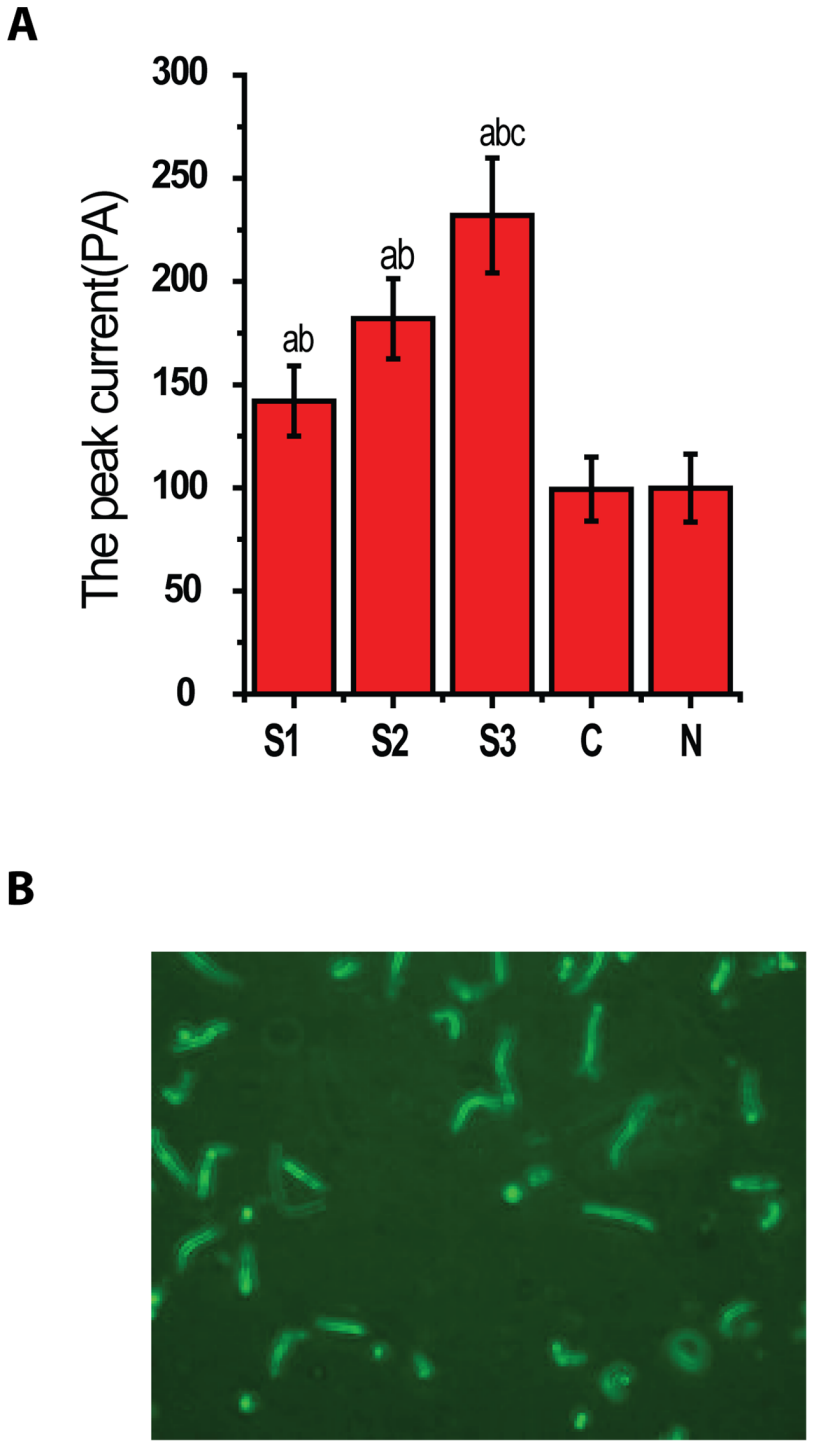
Characteristics of basilar artery smooth muscle cells. The graph A shows no significant difference for the membrane capacitance of BA smooth muscle cells in all group(P>0.05, n = 8/group).The graph B showed a representative image of smooth muscle cells isolated from the BA.

Single smooth muscle cell was isolated from the basilar arteries in each group. There was no significant difference in cellular morphology among all the groups, and there was no significant difference for the numbers of cells isolated from all groups (average number was about 40 cells in each high power field, P>0.05). There was no significant difference in cell capacitance among all the groups (Cm from 12.92±3.75 F to15.33±3.33, P>0.05) (Fig. 1).

### Characteristic currents evoked from different holding potentials

**Figure 2:**
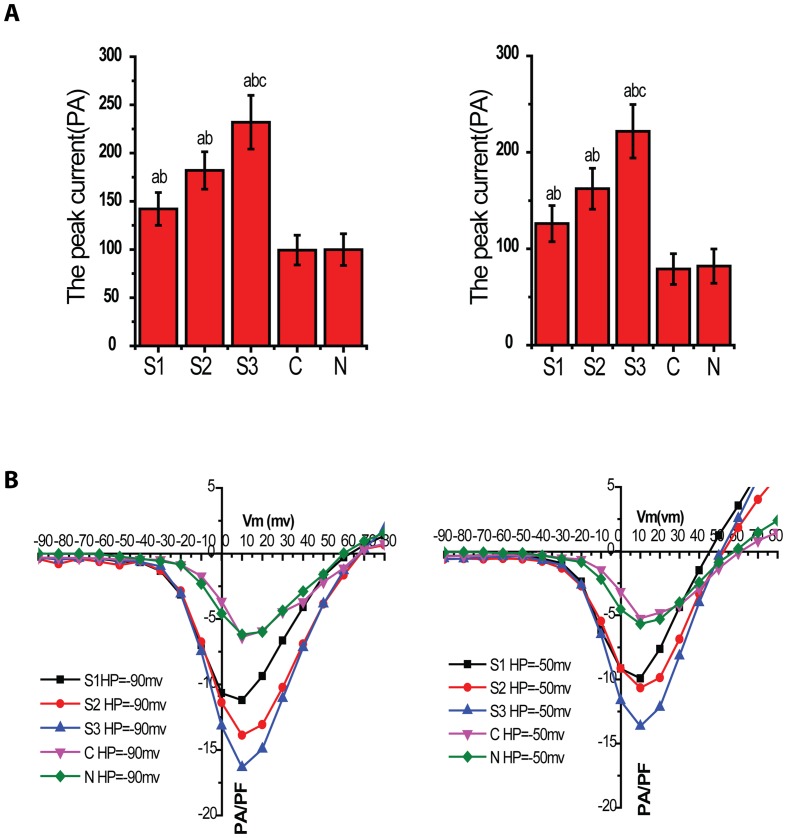
Activation curve of VDCCs in rabbit basilar artery smooth muscle cells after SAH. The graph A and B showed I-V plot at HP of −90mv and −50mv respectively. The ordinate was currents density (pA/pF) and the abscissa was voltage (mV). The two I-V plots showed the current density of VDCCs in S1–S3 group, which was bigger than that in N and C group at HP of −90mV and −50mV (P<0.05, n = 8/group). The current density in S3 group was bigger than those in S1 and S2 group (P<0.05, n = 8/group).The current density was not significantly different between C and N group (P>0.05, n = 8/group).

Calcium currents were recorded in isolation by excluding sodium and potassium ions rom both extracellular and intracellular solutions. The current density voltage(I-V) plot showed that the current of VDCCs in 24, 48 and 72 hour post SAH group was higher than that of normal (N) and sham group (C) at holding potential (HP) of −90mv and −50mv(P<0.05) (Fig. 2).The current in 72 hour post SAH group (S3) was significantly higher than that of 24 and 48 hour post SAH group (P<0.05).The current was not statistically significant between C and N group (P>0.05). Each of these families of traces shows that the peak current increased in each voltage step from −50mV to +50mV.

### Activation characteristics of VDCCs at HP of −90mv and −50mv

**Figure 3:**
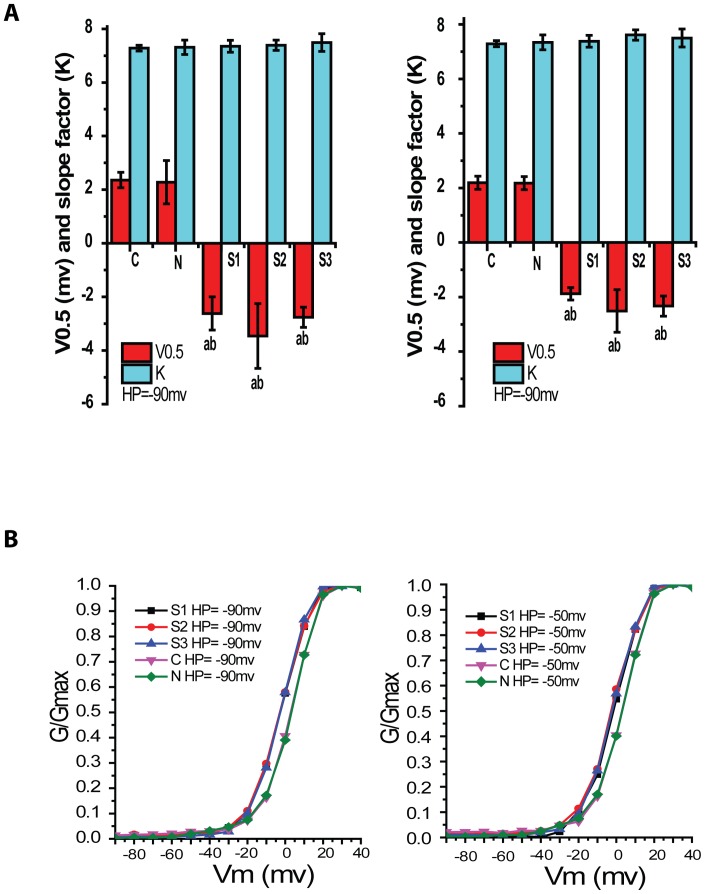
Activation characteristics of VDCCs in rabbit basilar artery smooth muscle cells after SAH. Graph A and B showed steady-state activation curves at HP of −90mV and −50mV respectively. The abscissa was voltage (mV) and the ordinate was relative conductivity (G). Compared with C and N group, steady-state activation curves of S1–S3 groups at HP of −90mV and −50mV shifted to the left (p<0.05, n = 8/group).

Based on I-V plot, the reversal voltages (V_rev_) of each group and its test potential (TP) were obtained from the rising part above the lowest point of linear fitting curve. The maximal conductance (G_max_) was calculated from the following equation, G = I/(Vm-Vrev), Where I is I_Ba_^2+^ peak value of TP, Vm is the testing voltage. G_max_ was then calculated and used to standardize each conductance to obtain relative membrane conductance (G/Gmax), then the activation curve of each group was plotted by origin 8.6 (Fig. 3). Voltage of half-activation (V_0.5_) and slope factor (k) for activation data were obtained through Boltzmann equation and described in Table 2.

**Table 2:** The voltage of half-activation (V_0.5_) and slope factor (K) at HP of −90mV and −50Mv.

Group	Cell number	HP = −90mV		HP = −50mV	
		V_0.5_	K	V_0.5_	K
C	8	2.36±0.29	7.28±0.59	2.19±0.24	7.29±0.11
N	8	2.28±0.81	7.31±0.19	2.18±0.24	7.34±0.27
S1	8	−2.62±0.62^ ab^	7.35±0.15	−1.88±0.23^ ab^	7.38±0.22
S2	8	−3.46±1.21^ ab^	7.39±0.36	−2.51±0.78^ ab^	7.61±0.19
S3	8	−2.76±0.38^ ab^	7.49±0.30	−2.33±0.37^ ab^	7.50±0.33

a P<0.05 vs C group,^ b^P<0.05 vs N group.

Compared with that of C group, Steady-state activation curves of S1–S3 groups at HP of −90mv and −50mv shifted to the left. There was no significant difference between C group and N group. Compared with that of C group, the voltage of half-activation of S1–S3 groups at HP of −90mv and −50mv was smaller (P<0.01) , however, the value of slope factor had no difference in every group. The curves indicated more Ca^++^ channels available to allow Ca^++^ entry after SAH and that will allow further Ca^++^ increase, but there is no change in channel opening rate, which might probably be the important factor causing vasospasm after SAH.

### Inactivation characteristics of VDCCs at HP of −90mV and −50mV

**Figure 4:**
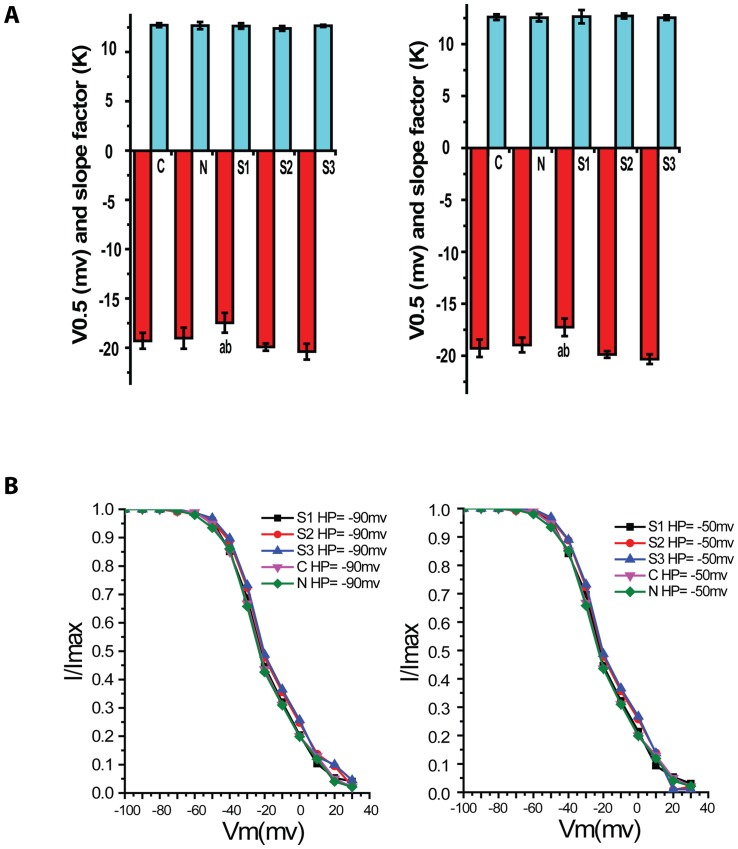
Voltage-dependent Ca+2 channel currents in rabbit basilar artery smooth muscle cells after SAH. The graph A and B showed steady-state inactivation curves at HP of −90mV and −50mV respectively. The abscissa was voltage (mV) and the ordinate was relative conductivity (G). Compared with C group, steady-state inactivation curves of S1–S3 groups at HP of −90mV and −50mV shifted to the right, but it had only significant difference in S3 group (P<0.05, n = 8/group).

Calcium current amplitude of whole cell induced by double pulse protocol was measured at TP = +30mV. Normalization to the evoked current in different pulse conditions was done to obtain Imax. Then relative current amplitude was obtained by I/Imax. And steady inactivation curve of groups were finally plotted (Fig. 4). Voltage of half-activation (V_0.5_) and slope factor for (k) activation data were obtained through Boltzmann equation (Table 3).

**Table 3:** The voltage of half-inactivation (V_0.5_)and slope factor(k) at HP of −90mv and −50mV.

Group	Cell number	HP = −90mV		HP = −50mV	
		V_0.5_	K	V_0.5_	K
C	8	−19.92±0.38	12.39±0.25	−19.88±0.33	12.71±0.24
N	8	−20.39±0.80	12.65±0.11	−20.32±0.47	12.54±0.22
S1	8	−19.29±0.80	12.72±0.21	−19.28±0.84	12.60±0.27
S2	8	−19.02±1.07	12.68±0.37	−18.96±0.71	12.54±0.36
S3	8	−17.46±1.00^ ab^	12.64±0.29	−17.26±0.85^ ab^	12.64±0.64

a P<0.05 Vs C group, ^b^P<0.05 vs N group.

The curve suggested that there was no significant shift in inactivation curves of S1 and S2 groups at HP of −90mV and −50mV, no evident change in half activation voltage. Compared with C group, the inactivation curve of S3 group shifted to the right and its voltage of half-inactivation was higher (P<0.05). The slope factor was no obvious change in every group. The curve suggested that the activated channel will totally lose its activity under higher voltage.

### Effect of Nifedipine on VDCCs in smooth muscle cell of rabbit basilar artery

**Figure 5:**
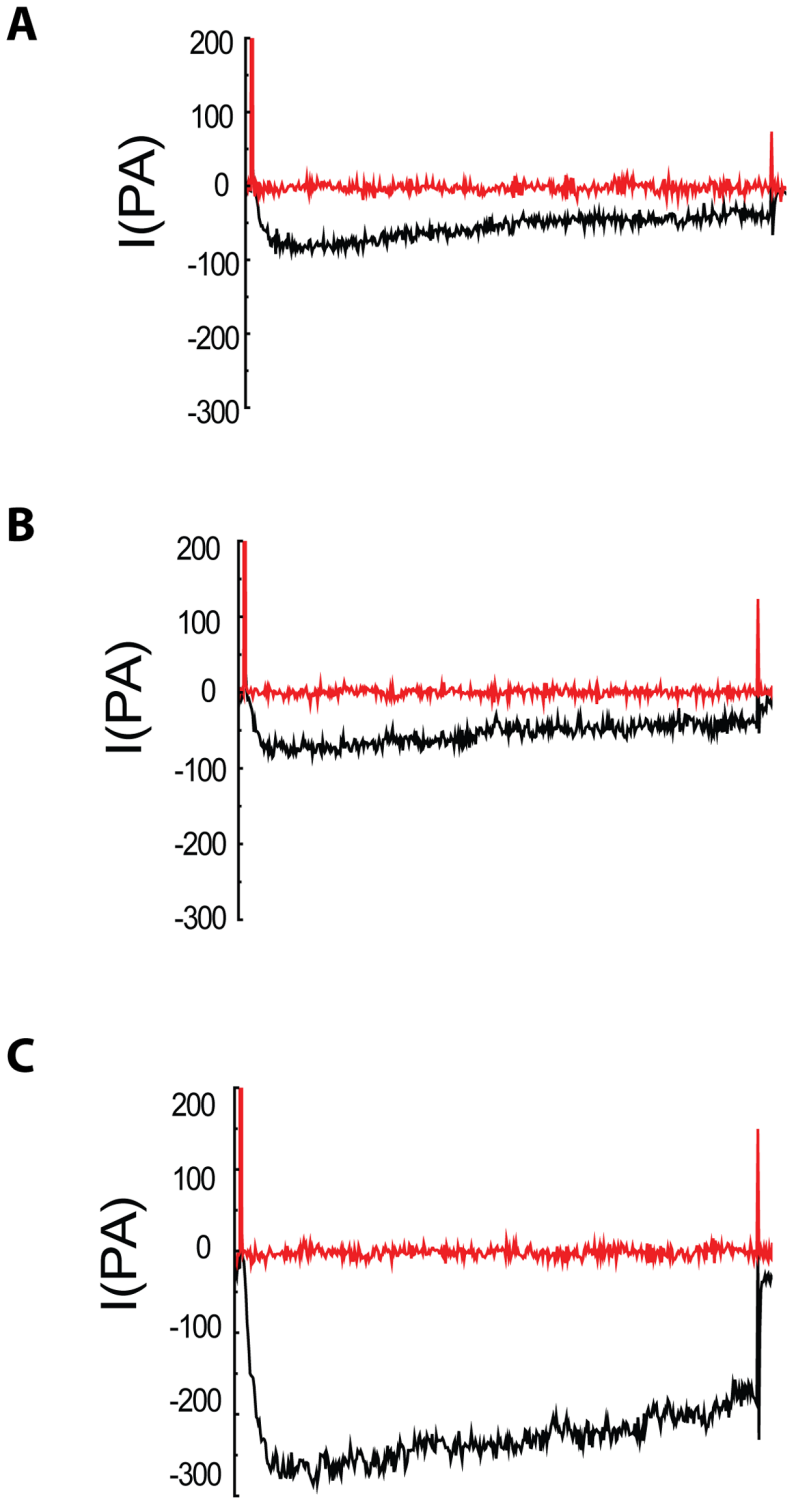
Analyses of current–voltage relationship for Ca+2 channel currents in rabbit basilar artery smooth muscle cells after SAH. The graph A , B and C showed the inhibitory effect of nifedipine on the currents of VDCCs in control (C) group, sham (N) group and SAH group respectively. The results confirmed nifedipine could inhibit the currents of VDCCs in every group.

Nifedipine is a selective L-type VDCC blocker. In order to validate whether the current was mediated by calcium channel, L-VDCC antagonist of nifedipine was added in cell solution, and the results suggested that nifedipine could effectively inhibit channel current, and inhibit completely channel current in every group at HP of −90mV and −50mV (Fig. 5). These results suggest that calcium channel current is L-Type VDCCs.

### L-Type VDCCs were predominantly expressed in human brain vascular smooth muscle cells (HBSMCs)

**Figure 6:**
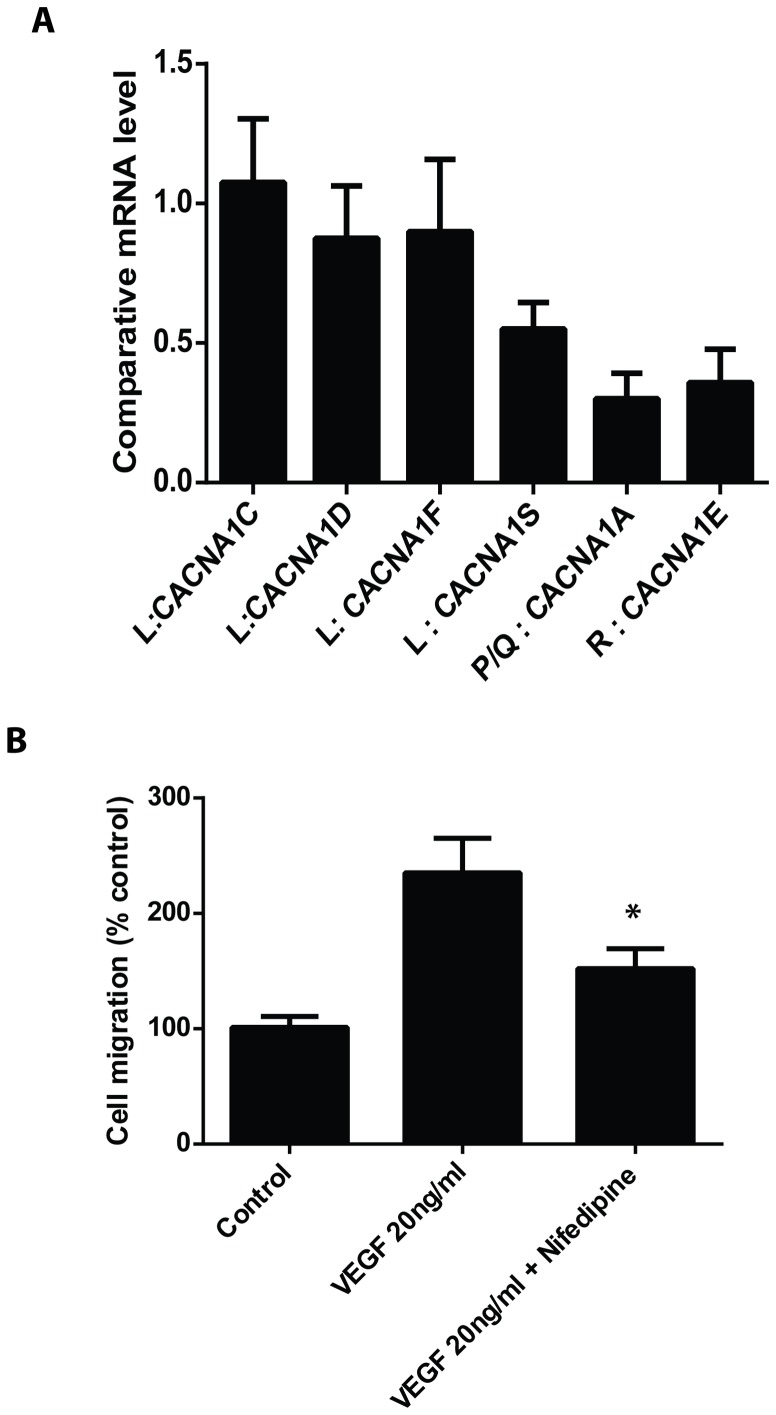
Expression of VDCCs in human brain vascular smooth muscle cells. **A,** Expression of different types of VDCCs were detected by real-time RT-PCR. **B,** Wound healing assay was performed with or without nifedipine. *p<0.05 versus VEGF treated group.

To examine whether VDCCs are present in human brain vascular smooth muscle cells, real-time RT-PCR was performed to detect mRNA levels in the cells. As shown in Figure 6A, the L type Ca2^++^ channel is predominantly present in HBSMCs. VEGF induced cell migration of HBSMC was inhibited by nefidipine (Figure 6B), suggesting that L type Ca2^++^ channels are important for HBSMC functioning.

## Discussion

In this study, we used a rabbit model of SAH to examine the changes of VDCCs in vascular smooth muscle cells of the basilar artery. The enzyme concentration for cell isolation, digestion time, and integrity of the isolated cells were optimized to meet the experimental needs. The technology of whole-cell patch clamp was harnessed to study the alteration in VDCC currents. VDCCs current in 24–72 hours group after SAH was higher than those of control group and sham group. However, there was no significant difference in VDCC current between control group and sham group. I-V plot indicated that current density of S1–S3 group was obviously higher than that of C group and N group in different HP conditions with membrane potential between −20mv to 10mv, and such increase was more significant in response to prolonged time, suggesting there were more Ca2^+^ influx through VDCCs and further cause stronger contraction of muscle smooth cells. Kovacic et al [26] found the presence of CVS 48 hours after SAH in a rabbit SAH model and it lasted for several days. Lin et al [27] demonstrated CVS began at 12 h after SAH in rats model and lasted for 3 days. Mizuno et al [28] demonstrated the presence of BA spasm through single blood injection into the ventral cisterna magna through a microcatheter by angiography. All the findings were consistent with our experimental results.

In order to identify L-type VDCCs current and T-type VDCCs current in the experiments, HP was set up to −90mv and −50mv because L-type VDCCs is only activated by high voltage (activation HP between −30mv and −40mv) and T-type VDCCs is activated by low voltage (activation pressure about −60mv) [15] [29]. In this study, the activation voltage of VDCCs was from −30mv to −40mv, and nifedipine, a L-type VDCCs antagonist could completely inhibit the Ca^++^ channel current. These results suggested that Ca^++^ channel current is L-Type VDCCs in this study. It indicated the L-type Ca^++^ channel current might play important effect at early stage of SAH.

Compared with control and sham group, the steady-state activation curves in 24, 48 and 72 hours groups shifted to the left at HP of −90mv and −50mv and had lower half-activation voltage. This indicated low up-threshold stimulation could open the VDCCs in smooth muscle cells (SMCs) of basilar artery and cause a large of Ca^++^ influx after SAH. It maybe had relation with the change of VDCCs function after SAH [16] [18] [30]. The influx of calcium ion caused contraction of smooth muscle cells in basilar artery and cerebral vasospasm. The studies discovered it would increase intracellular calcium ion level in cerebrovascular SMCs and angiotasis after SAH and nifedipine could decrease the change [30], [31], [32].The results indicated that VDCCs had important effect on the cerebral vasospasm after SAH.

Compared with C group, the steady-state inactivation curve in 72 hour (S3) group shifted to the right and its voltage of half-inactivation was higher at HP of −90mv and −50mv (P<0.05).There was no significant difference for half-inactivation voltage between S1–S2 and C group (P>0.05). The slope factor was no obvious change in every group. The results suggested that the activated channel would totally lose its activity under high inactivation voltage. It would be an important factor for sustained cerebral vasospasm.

From the above, the VDCCs in SMCs of basilar artery had a lower voltage threshold to open and a higher voltage threshold to close in S1–S3 group after SAH than that in N and C group. In other words, the VDCCs could be opened with low activation voltage and closed with high inactivation voltage after SAH. Therefore, a large of Ca^++^ would come into SMCs and lead to increase intracellular Ca^++^. Eventually, it would lead to the contraction of SMCs and cerebral vasospasm.

The rabbit model of SAH utilized in this study is a well-established SAH experimental model, in which blood clot formation around BA contributes to the development of CVS in early period after SAH. Previous results based on this animal model were in accordance with cerebral vasospasm of clinic patients [20],[32],[33],[34],[35]. In our study, we demonstrated that human brain vascular smooth muscle cells (HBSMCs) indeed have expression of VDCCs, with the highest levels of the L-Type VDCCs. The L type Ca2^++^ channels are important for HBSMC functioning since VEGF induced cell migration of HBSMC was inhibited by nefidipine. These results suggest that the altered VDCC currents observed in this study may have important relevance to the clinical settings of human SAH.

The key findings of the changes occurred in basilar artery smooth muscle cells at 72 hours after SAH is of significant interest to therapeutic intervention. The change of VDCCs in the structure and function after SAH could cause the open of VDCCs and influx of calcium ion in SMCs of basilar artery. These results would lead to the contraction of SMCs and cerebral vasospasm. The VDCCs may play an important role for cerebral vasospasm after SAH. Since VDCCs are well known regulator in many cellular process including oxidative stress, mitochondrial function, and cell death, it would be important to explore whether these molecular mechanisms are involved in SAH-induced vasospasm. Further investigation in this direction would identify novel therapeutic targets at early stage of subarachnoid hemorrhage to fight against this deadly pathological condition.
